# BC-DUnet-based segmentation of fine cracks in bridges under a complex background

**DOI:** 10.1371/journal.pone.0265258

**Published:** 2022-03-15

**Authors:** Tao Liu, Liangji Zhang, Guoxiong Zhou, Weiwei Cai, Chuang Cai, Liujun Li

**Affiliations:** 1 College of Civil Engineering, Central South University of Forestry and Technology, Changsha, Hunan, China; 2 College of Computer & Information Engineering, Central South University of Forestry and Technology, Changsha, Hunan, China; 3 Department of Civil, Architectural and Environmental Engineering, University of Missouri-Rolla, Rolla, MO, United States of America; Al Mansour University College-Baghdad-Iraq, IRAQ

## Abstract

Crack is the external expression form of potential safety risks in bridge construction. Currently, automatic detection and segmentation of bridge cracks remains the top priority of civil engineers. With the development of image segmentation techniques based on convolutional neural networks, new opportunities emerge in bridge crack detection. Traditional bridge crack detection methods are vulnerable to complex background and small cracks, which is difficult to achieve effective segmentation. This study presents a bridge crack segmentation method based on a densely connected U-Net network (BC-DUnet) with a background elimination module and cross-attention mechanism. First, a dense connected feature extraction model (DCFEM) integrating the advantages of DenseNet is proposed, which can effectively enhance the main feature information of small cracks. Second, the background elimination module (BEM) is proposed, which can filter the excess information by assigning different weights to retain the main feature information of the crack. Finally, a cross-attention mechanism (CAM) is proposed to enhance the capture of long-term dependent information and further improve the pixel-level representation of the model. Finally, 98.18% of the Pixel Accuracy was obtained by comparing experiments with traditional networks such as FCN and Unet, and the IOU value was increased by 14.12% and 4.04% over FCN and Unet, respectively. In our non-traditional networks such as HU-ResNet and F U N-4s, SAM-DUnet has better and higher accuracy and generalization is not prone to overfitting. The BC-DUnet network proposed here can eliminate the influence of complex background on the segmentation accuracy of bridge cracks, improve the detection efficiency of bridge cracks, reduce the detection cost, and have practical application value.

## 1. Introduction

With the development of building defect detection technology, image processing technology has been increasingly applied to the recognition and classification of building defects. Studies have been conducted on road crack detection, house crack detection, and other fields. These topics have become the key points of contemporary age building defect detection studies, and remarkable achievements have been achieved to some extent. A bridge is one of the key components of a transportation building system, and it is extremely important to conduct periodic detection of bridges. The calculation of the related indexes of bridge cracks is an important indicator of bridge detection [1]. For example, if the cracks are smaller than 0.2 mm, they may be prone to interference by surrounding obstacles, resulting in potential safety hazards of detection omission. Therefore, a quick and accurate recognition and detection of fine cracks in bridges under complex backgrounds and timely implementation of appropriate maintenance measures are of great significance for the structural safety of bridges and traffic safety.

Cracks are one of the most common bridge defects and are often distributed irregularly; thus a large number of manual photography and sampling tasks are required [2]. Presently, the collected data such as crack images are mainly analyzed and judged manually; however, manual analysis is subject to low work efficiency, high labor intensity, poor reproducibility, high labor cost, low accuracy, and other defects [3]. In addition, during bridge detection at the bottom, the workers usually have limited observation fields, which may cause certain problems such as incomplete investigation and omission of detection results. In early bridge crack recognition models, the attribute of cracks captured in the images was described by the traditional extraction of global low-level features such as texture, shape, and edge. However, these models were easily affected by complex road conditions such as the size of pavement texture particles, road and bridge joints, and marking edges. Therefore, their application was greatly limited. Recently, digital image processing technology, which is mostly limited to two-dimensional grayscale image analysis technology, has been employed for asphalt pavement crack recognition [4]. However, because of the low signal-to-noise ratio of two-dimensional images, the target information cannot be accurately extracted; therefore the application effect is unsatisfactory. Presently, deep learning has attracted considerable attention. Compared to shallow models, deep learning is advantageous for feature extraction and recognition. However, classical deep learning models are mainly classification or recognition models for datasets with large sizes and overall targets, such as AlexNet [5], GoogLeNet [6]. Furthermore, it also includes faster models like R-CNN, etc [7]. Because of the different shapes and linear topological structure of the cracks, if these deep learning models are directly applied to bridge crack detection, they will be prone to detection omission and misjudgment.

Considering bridge crack detection, cracks are easily affected by complex road conditions, including the size of pavement texture particles, marking edges, and other interference information, which will result in difficulty in segmentation and effective recognition. Therefore, based on these features, BC-DUnet is proposed in this study, which can better achieve small target segmentation of fine cracks in bridges under complex backgrounds. The contributions of this study are summarized as follows.

1. Based on Densnet’s advantage of enhancing feature transfer, a densely connected feature extraction module is proposed. This module can effectively make use of detailed features, alleviate the vanishing gradient problem of models, and improve the segmentation ability for the main feature information of fine cracks in bridges.
2. A background elimination module is proposed to filter redundant information by assigning different weights. This can retain complete images of fine cracks in bridges by eliminating the complex background of fine cracks, improving the module processing accuracy.
3. A cross attention mechanism that can assign weighting coefficients to each row and column based on the horizontal and vertical attention mechanisms, respectively, is proposed.It can also enlarge the differences between features by weight multiplication and maximum weight matching policies, improving the accuracy of segmentation of the model.
4. The experimental results show that compared to deep neural networks, the proposed network shows significantly higher performance, accuracy, and versatility, making bridge detection and monitoring work efficient, inexpensive, and ultimately automated.

## 2. Related studies

Recently, considering the development of the traffic construction industry, an increasing number of experts have begun to explore identification of bridge defects quickly and effectively. A number of different methods, such as digital image processing technology, target detection technology, and neural networks, can be applied to crack detection and recognition. This can significantly improve the efficiency of manual or semi-automatic defect detection. Yuan et al. [8] adopted the gray threshold segmentation and interference elimination methods by combining binary and grayscale images to identify cracks. However, this method is unsuitable in cases where cracks and background differences are not obvious. Kamaliardakani et al. [9] employed classical gray threshold segmentation for identification of cracks; however, this method is easily affected by lighting shadows on road surfaces. Ma et al. [10] applied a non-subsampled contourlet transform combined with an image morphological method and median filtering to realize crack detection; however, this method is computationally expensive. Li et al. [11] proposed a method based on the dual-tree complex wavelet transform, which ensured accuracy and improved the anti-interference ability. Qu et al. [12] proposed a pavement crack extraction algorithm that combined texture feature fusion and saliency detection and achieved high accuracy and recall rates. However, this algorithm achieves an extremely low success rate of modeling recognition for nonlinear data, which is unsatisfactory for complex bridge crack detection. Lee et al. [13] proposed a pavement crack segmentation and recognition algorithm based on a BP neural network, which segmented the original image into numerous sub-images and performed threshold segmentation. However, it is defective because it can only recognize transverse and longitudinal cracks, and it is not ideal for identifying small cracks such as fine cracks.

The literature shows that although some cracks can be recognized and extracted using traditional image processing to a certain extent, owing to the particularity of a bridge environment, the models are confronted with certain complex background problems such as rough surface, bulge, sinking and stain. In addition, pictures collected in different bridge environments impose an adverse impact on the test results; therefore, it is difficult to guarantee the robustness of these methods. Moreover, the aforementioned algorithms can only achieve good accuracy in small samples. When the crack shape is not obvious or the crack is small, regardless of whether the traditional feature extraction is possible, the detection of cracks cannot be guaranteed.

Deep learning can be applied for effective automatic crack detection, to improve the speed and accuracy of crack recognition, further enhance the crack analysis efficiency, and reduce workload. Zhang et al. [14] proposed a convolutional neural network (CNN) model for determining whether there are cracks in small two-dimensional pavement images and compared it to the traditional machine learning methods, SVM and Boost. The results show that the CNN is more suitable for crack detection; however its accuracy is only 86.96%, which cannot meet actual engineering requirements. In addition, Zhang et al. [15] and Chen et al. designed a crack detection method based on sliding window scanning; however, this method is slow [16]. Cha et al. [17] proposed a crack detection method that combined a CNN model with a sliding window, which reduced the influence of local factors in the image. The methods in the aforementioned literature have revealed good performance in crack applications, which is to say, they can locate cracks in images; however, they cannot detect cracks pixel-by-pixel. Using pixel-level prediction techniques, geometric features of cracks such as shape, direction, length, and width can be acquired. These are crucial for accurately evaluating pavement conditions and making decisions on pavement maintenance. Zhu et al. [18] employed an improved U-Net to detect pixel-level bridge crack images with small samples. Liu et al. [19] adopted the U-Net method for concrete crack detection. Compared to the FCN, this method achieves high efficiency, good robustness, and better accuracy. Nevertheless, certain problems such as loss of details and false detections still exist. Xu et al. [20] based on U-Net, adopted the pre-trained ResNet34 as an encoder and added an attention mechanism module to improve the accuracy of concrete crack detection. However, using this method, it is difficult to effectively extract the main feature information under a complex background.

Considering actual bridge crack images, cracks are easily affected by complex backgrounds, such as rough surfaces, bulges, sinks, and stains. The methods described in the aforementioned literature cannot effectively segment the main features of cracks. Therefore, it is necessary to for design an effective segmentation network method to acquire feature information that is conducive for crack segmentation under complex backgrounds to improve the accuracy of segmentation and generalization ability of the network.

## 3. Materials and methods

### 3.1 Data acquisition

**Fig 1:**
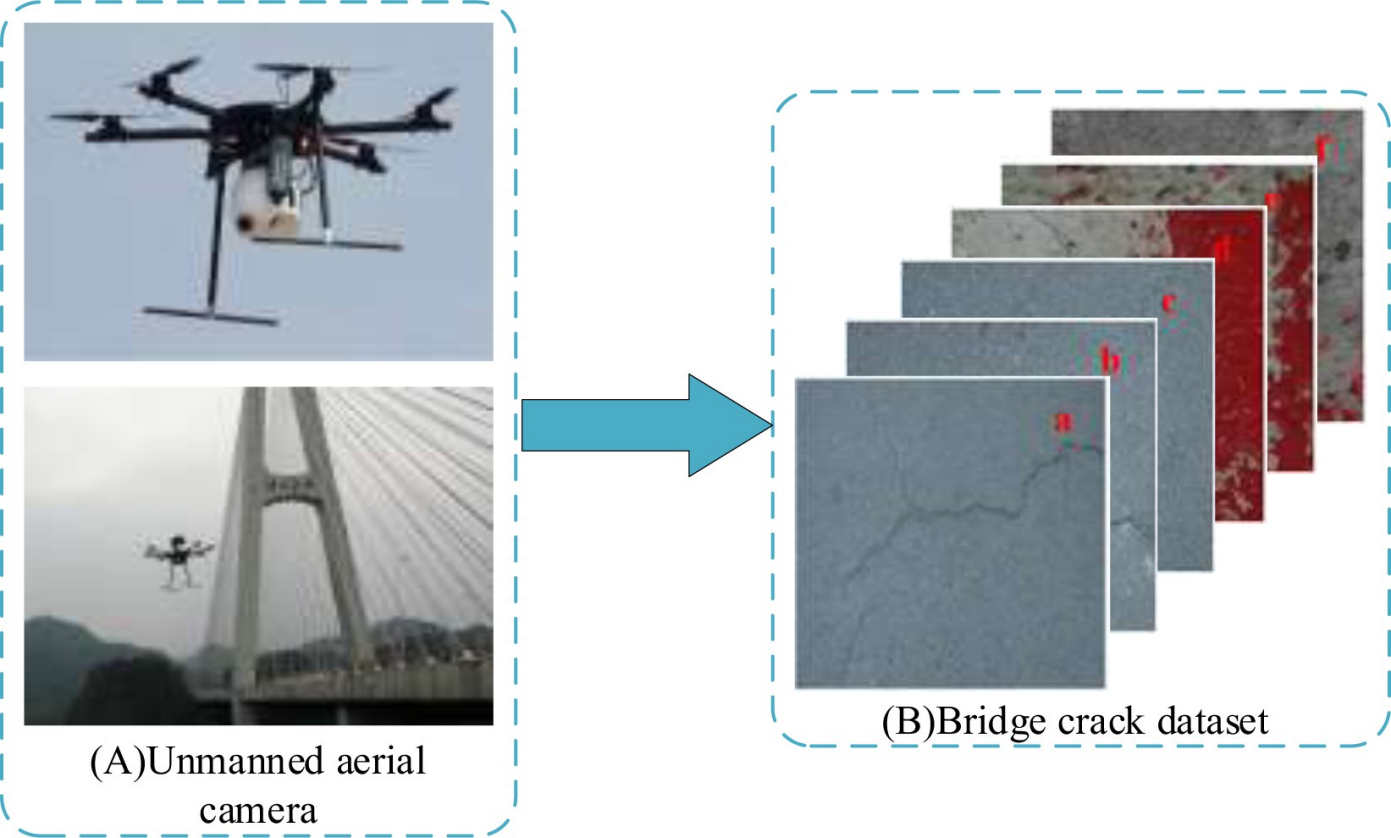
Crack collection mechanism.

Based on Li et al.’s [21] study and considering that there is no unified bridge crack database, a dataset on the website https://github.com/tjdxxhy/crack-detection was employed to enrich the bridge crack data and meet the experimental requirements. The dataset included 6,070 bridge crack images with a resolution of 224 × 224, of which 2,750 images were randomly selected. Using this dataset, the bridge crack collection mechanism, as shown in Fig 1, is constructed in this study to acquire crack images; this will enrich the bridge crack images. In addition, 500 pictures were captured by a CCD industrial camera (Basler acA1300-30gm) mounted on a UAV with a resolution of 4896 × 3672 and were saved in JPG. They were mostly crack pictures under complex backgrounds (such as crack pictures with rough surfaces, bulges, sinking, stains, etc.). These images were different owing to different shooting conditions; nevertheless, they were all input with a uniform size of 224 × 224. Furthermore, these crack images were manually labeled by Labelme, a deep learning image labeling tool, with crack and background pixels labeled in white and black, respectively, to enable the storage of image information in JSON. Finally, 3,250 images were acquired, and the dataset was divided into training and test sets with 2,600 and 650 images, respectively, at a ratio of 8:2 for model training and testing.

### 3.2 BC-DUnet

**Fig 2:**
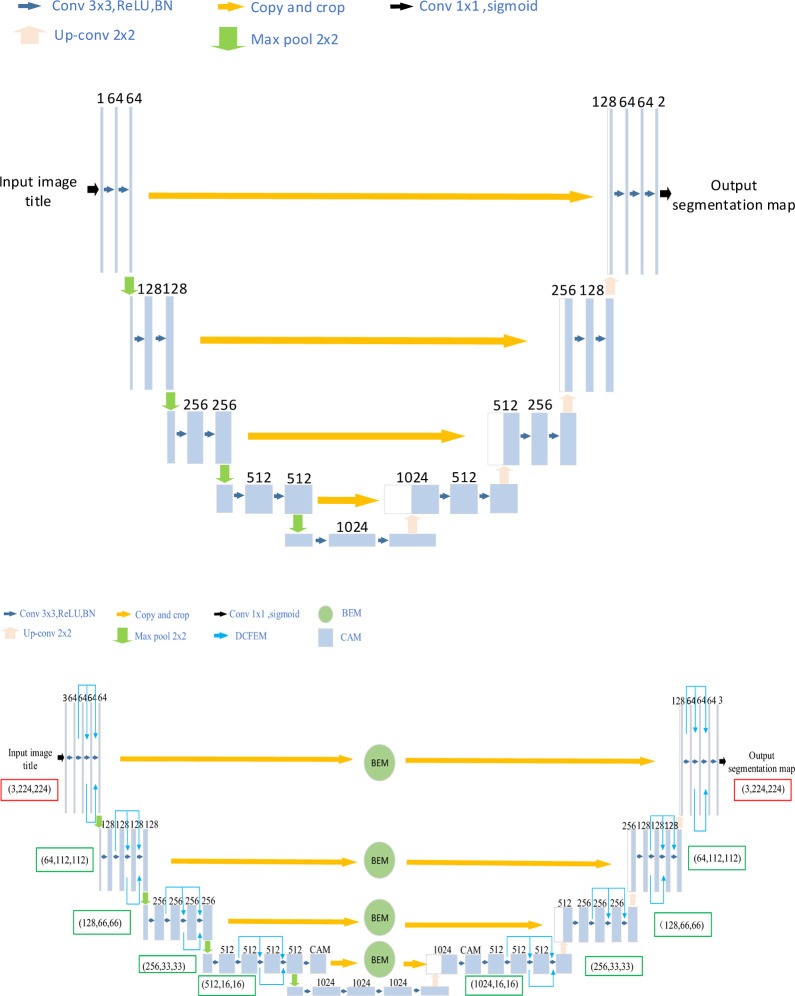
Network structure diagrams of U-Net and BC-DUnet. (a) U-Net network structure. (b) BC-DUnet network structure.

The overall framework of the BC-DUnet network model proposed in this study is presented in Fig 2(B). Based on the U-Net model shown in Fig 2(A), BC-DUnet introduces a densely connected feature extraction module, background elimination module, and cross attention mechanism. Considering this model, a densely connected feature extraction module is employed instead of the ordinary convolution layer. This can effectively enhance the main feature information of fine cracks. Because cracks are easily affected by complex backgrounds such as rough surfaces, bulges, sinking, and stains, the background elimination module can retain detailed information of cracks while removing the complex background information. In addition, the cross attention mechanism can assign weighting coefficients to each row and column, using horizontal and vertical attention mechanisms, respectively. It can also expand the differences between features using the weight multiplication and maximum weight matching policies. This further strengthens the main feature ability of cracks and improves the accuracy of segmentation. The parameters of each layer of the model are listed in Table 1. The densely connected feature extraction module, background elimination module, and cross attention mechanism are introduced in detail in the subsequent sections.

**Table 1:** Network parameters.

Layer	Parameter	Follow-up actions
Input	224×224×3	
Down-sampling 1	64 convolution filters (3×3), 1 strides,1 padding, BN+Relu (conv1)	
	64 convolution filters (3×3), 1 strides,1 padding, BN+Relu (conv2)	DCFEM1+conv2
	64 convolution filters (3×3), 1 strides,1 padding, BN+Relu (conv3)	DCFEM 1+ DCFEM 2+conv3
	64 convolution filters (3×3), 1 strides, 1 padding, BN+Relu(conv4)	
	Max pooling (2×2), 2 strides	
Down-sampling 2	128 convolution filters (3×3), 1 strides,1 padding, BN+Relu (conv5)	
	128 convolution filters (3×3), 1 strides,1 padding, BN+Relu (conv6)	DCFEM 5+conv6
	128 convolution filters (3×3), 1 strides,1 padding, BN+Relu (conv7)	DCFEM 5+ DCFEM 6+conv7
	128 convolution filters (3×3), 1 strides,1 padding, BN+Relu (conv8)	
	Max pooling (2×2), 2 strides	
Down-sampling 3	256 convolution filters (3×3), 1 strides,1 padding, BN+Relu (conv9)	
	256 convolution filters (3×3), 1 strides,1 padding, BN+Relu (conv10)	DCFEM 9+conv10
	256 convolution filters (3×3), 1 strides,1 padding, BN+Relu (conv11)	DCFEM 9+ DCFEM 10+conv11
	256 convolution filters (3×3), 1 strides,1 padding, BN+Relu (conv12)	
	Max pooling (2×2), 2 strides	
Down-sampling 4	512 convolution filters (3×3), 1 strides,1 padding, BN+Relu (conv13)	
	512 convolution filters (3×3), 1 strides,1 padding, BN+Relu (conv14)	DCFEM 13+conv14
	512 convolution filters (3×3), 1 strides,1 padding, BN+Relu (conv15)	DCFEM 13+ DCFEM14+conv15
	512 convolution filters (3×3), 1 strides,1 padding, BN+Relu (conv16)	
	Max pooling (2×2), 2 strides	
Down-sampling 5	1024 convolution filters (3×3), 1 strides,1 padding, BN+Relu (conv17)	
	1024 convolution filters (3×3), 1 strides,1 padding, BN+Relu (conv18)	DCFEM 17+conv18
	1024 convolution filters (3×3), 1 strides,1 padding, BN+Relu (conv19)	DCFEM 17+ DCFEM18+conv19
	1024 convolution filters (3×3), 1 strides,1 padding, BN+Relu (conv20)	
Up-sampling 1	512 Deconvolution filters (2×2), 2 strides (Deconv1)	
	Concat (CAM+BEM (conv16), Deconv1)	
	512 convolution filters (3×3), 1 strides, 1 padding, BN+Relu (conv21)	DCFEM 21+conv22
	512 convolution filters (3×3), 1 strides, 1 padding, BN+Relu (conv22)	DCFEM 21+ DCFEM22+conv23
	512 convolution filters (3×3), 1 strides, 1 padding, BN+Relu (conv23)	
	512 convolution filters (3×3), 1 strides, 1 padding, BN+Relu (conv24)	
Up-sampling 2	256 Deconvolution filters (2×2), 2 strides (Deconv 2)	
	Concat (CAM+BEM (conv1), Deconv2)	
	256 convolution filters (3×3), 1 strides, 1 padding, BN+Relu (conv25)	DCFEM 25+conv26
	256 convolution filters (3×3), 1 strides, 1 padding, BN+Relu (conv26)	DCFEM 25+ DCFEM26+conv27
	256 convolution filters (3×3), 1 strides, 1 padding, BN+Relu (conv27)	
	256 convolution filters (3×3), 1 strides, 1 padding, BN+Relu (conv28)	
Up-sampling 3	128 Deconvolution filters (2×2),2 strides (Deconv 3)	
	Concat (CAM+BEM (conv8), Deconv3)	
	128 convolution filters (3×3), 1 strides, 1 padding, BN+Relu (conv29)	DCFEM 29+conv30
	128 convolution filters (3×3), 1 strides, 1 padding, BN+Relu (conv30)	DCFEM 29+ DCFEM30+conv31
	128 convolution filters (3×3), 1 strides, 1 padding, BN+Relu (conv31)	
	128 convolution filters (3×3), 1 strides, 1 padding, BN+Relu (conv32)	
Up-sampling 4	64 Deconvolution filters (2×2), 2 strides (Deconv 4)	DCFEM 33+conv34
	Concat (CAM+BEM (conv4), Deconv4)	DCFEM 33+ DCFEM34+conv35
	64 convolution filters (3×3), 1 strides, 1 padding, BN+Relu (conv33)	
	64 convolution filters (3×3), 1 strides, 1 padding, BN+Relu (conv34)	
	64 convolution filters (3×3), 1 strides, 1 padding, BN+Relu (conv35)	
	64 convolution filters (3×3), 1 strides, 1 padding, BN+Relu (conv36)	
Output	3 convolution filters (3×3), 1 strides	64×64×1

#### 3.2.1 DCFEM

Generally, considering the deepening of network layers, the vanishing gradient problem becomes increasingly obvious. Therefore, it is necessary to first consider this problem arising from the network depth. Many studies have proposed solutions to this problem, such as Highway Network and Fractal Net [22, 23]. Although these algorithms have different network structures, the main concept is to establish a short connection from the lower layer to the higher layer of the network. DenseNet elaborates on this concept and directly connects all layers to ensure maximum information transfer between layers in the network [24].

**Fig 3:**
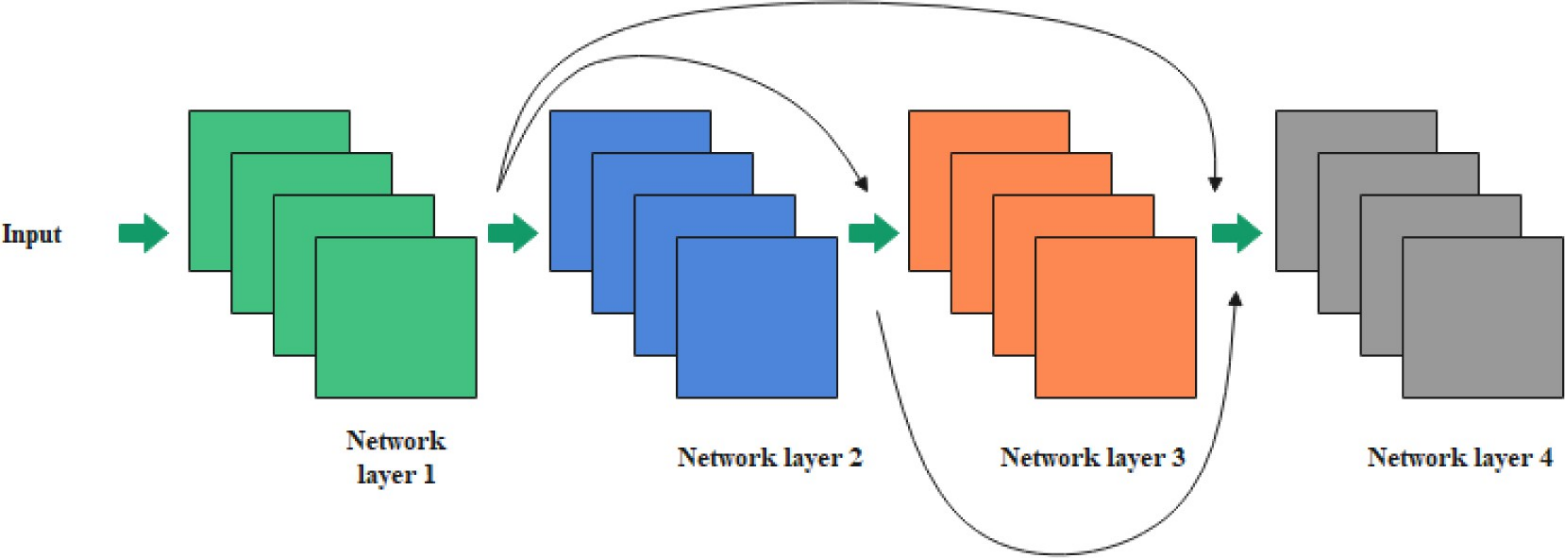
DCFEM.

The main network subject of the DCFEM is designed with four dense blocks, which are alternately connected in series. This backbone network, having established a connection relationship among different layers, can make full use of the features of fine cracks in bridges and ensure the validity of the feature map used in the network segmentation. The structure diagram is as shown in Fig 3.

The connection mechanism of the residual network is to perform a short connection of each layer with the previous one or two layers by adding each element. This can be expressed as follows:

$$x_{1}=H_{l}(x_{l-1})+x_{l-1}.$$ (1)

By contrast, the connection mechanism of DenseNet is to interconnect all layers. Each layer accepts all previous layers as input, and the feature map of each layer is fitted together in the channel dimension. This can be represented as follows:

$$x=H([x_{0},x_{1},\ldots ,x_{l-1}]),$$ (2)

where *x*_1_ refers to the output of the *l* layer, [*x*_0_,*x*_1_,…,*x*_*l*−1_] represents the splicing of feature maps from input layer to *1*−1 layer, and *H*_*l*_(.) indicates a nonlinear conversion function, which is an operation combining BN, ReLU, and convolutional layers. Regarding the DenseNet, each layer obtains an extra input from all the previous layers and continuously transfers its own feature mapping to all the subsequent layers. In addition, each layer of the DenseNet can receive feature information from all the previous layers to enable the transfer of feature information and gradient in the network. Furthermore, models can be trained in depth, and feature reuse is enhanced. The network structure of DenseNet is mainly composed of multiple dense blocks and transition layers, and feature maps in each dense block remain the same size, making dimensional splicing convenient. Assuming that each layer only generates *k* feature maps, according to the network design of DenseNet, the input dimension of the *l* layer can be expressed as follows:

$$k_{0}+(l-1)\times K,$$ (3)

Where *k*_0_ refers to the number of channels in the input layer. It can be observed from Eq (3) that if the number of channels in the feature map is not limited, the number of channels after splicing will increase. To reduce the number of feature maps, decrease the computing amount, and facilitate the fusion of channel features, a bottleneck layer is added to the dense block.

By combining DCFEM with U-net, detailed information on tiny cracks in the bridge can be effectively extracted.

#### 3.2.2 BEM

The bridge crack features are mainly embodied in the shape and texture of cracks; however, these cracks are easily affected by complex backgrounds such as rough surfaces, bulges, sinks, and stains. These are redundant information in images, and they interfere with the segmentation of bridge cracks. To effectively suppress the redundant information and retain the complete images of fine cracks in bridges, a background elimination module is designed in this study to improve the accuracy of the segmentation of fine cracks in bridges under complex backgrounds.

**Fig 4:**
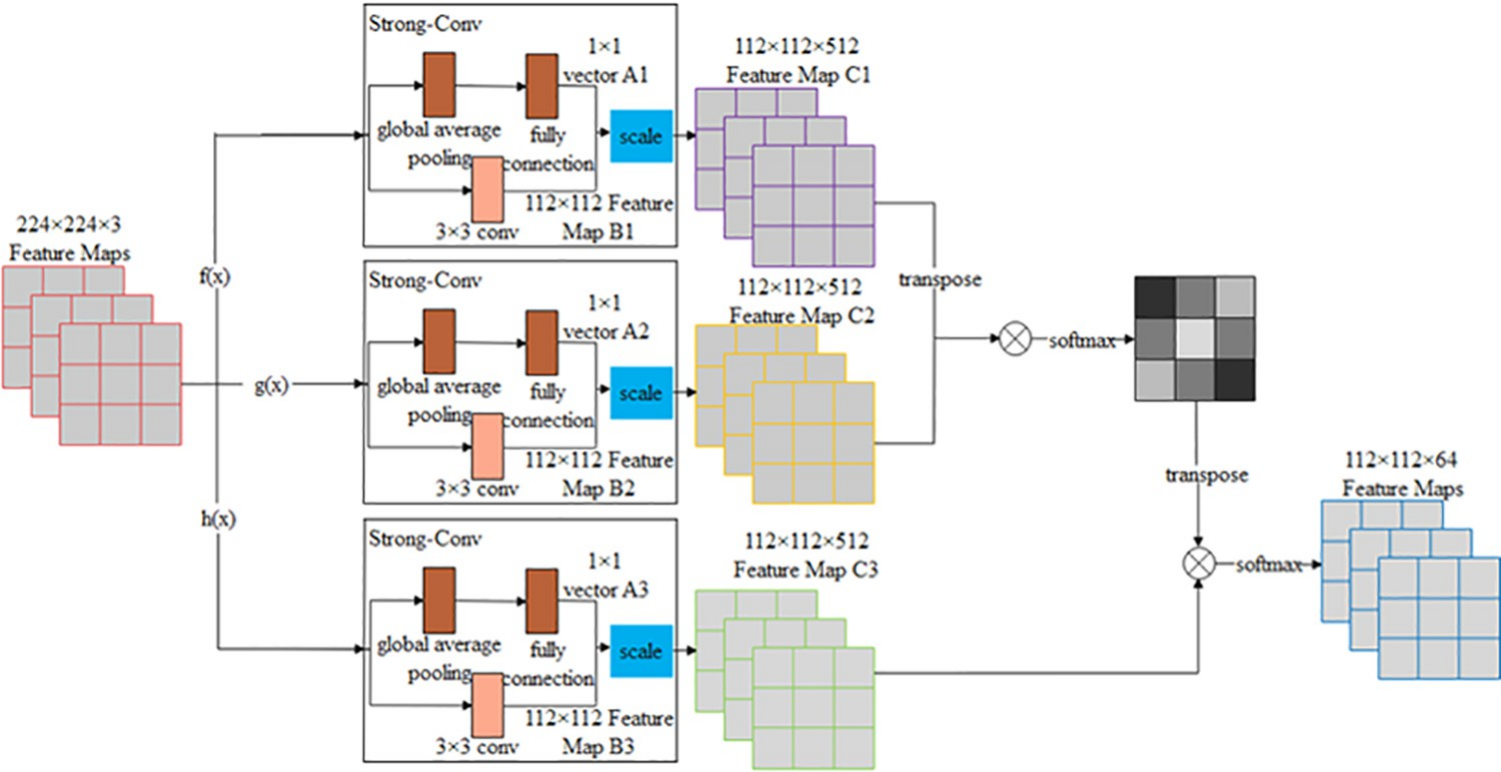
Structure of BEM.

Fig 4, shows the schematic diagram of the BEM with a 224 × 224 × 3 bridge crack feature map as the input. First, the feature map is mapped through three Strong-Conv module channels; the Strong-Conv module designed in this study consists of a 3 × 3 convolutional layer, global average pooling layer, fully connected layer, and linear computation. Considering this module, the channel and spatial features of the feature map input are learned. Regarding the global feature learning branch, the global average pooling and fully connected layers are employed to learn the correlation between channels of the feature map input, and a 1 × 1 vector Ai (i = 1,2,3) is obtained. Considering the other spatial learning branch, a 3 ×3 convolutional layer was applied to learn the spatial correlation of the feature map. Consequently, 112 × 112 feature maps Bi (i = 1,2,3) are acquired, and a 112 × 112 feature map Ci (i = 1,2,3) is achieved through feature fusion by linear operation, in which the sizes of feature maps C1 and C2 are 112 × 112 × 64, and C3 is 112 × 112 × 512. Thereafter, the transposed feature maps C1 and C2 are multiplied by the corresponding elements of the matrix, and a 64 × 512 attention map is obtained through Softmax. Finally, the transposed attention map and feature map C3 are multiplied by the corresponding elements of the matrix, and a 112 × 112 × 64 feature map is output through Softmax. The design of the Strong-Conv module further strengthens the attention to the information of bridge crack characteristics.

#### 3.2.3 CAM

Limited by the size of the convolution kernel of the traditional convolutional network, only the relationship between the local regions can be captured. Therefore, a cross attention mechanism is proposed in this study. This mechanism can balance the large-scale structure and computational statistical efficiency, assign two horizontal and vertical weighting coefficients to each feature, expand the differences between characteristics by weight multiplication and maximum weight matching policies, and enhance the capture of long-term dependency information by the model. Therefore, it enhances the pixel-level representation ability and highlights the main feature information of fine cracks.

**Fig 5:**
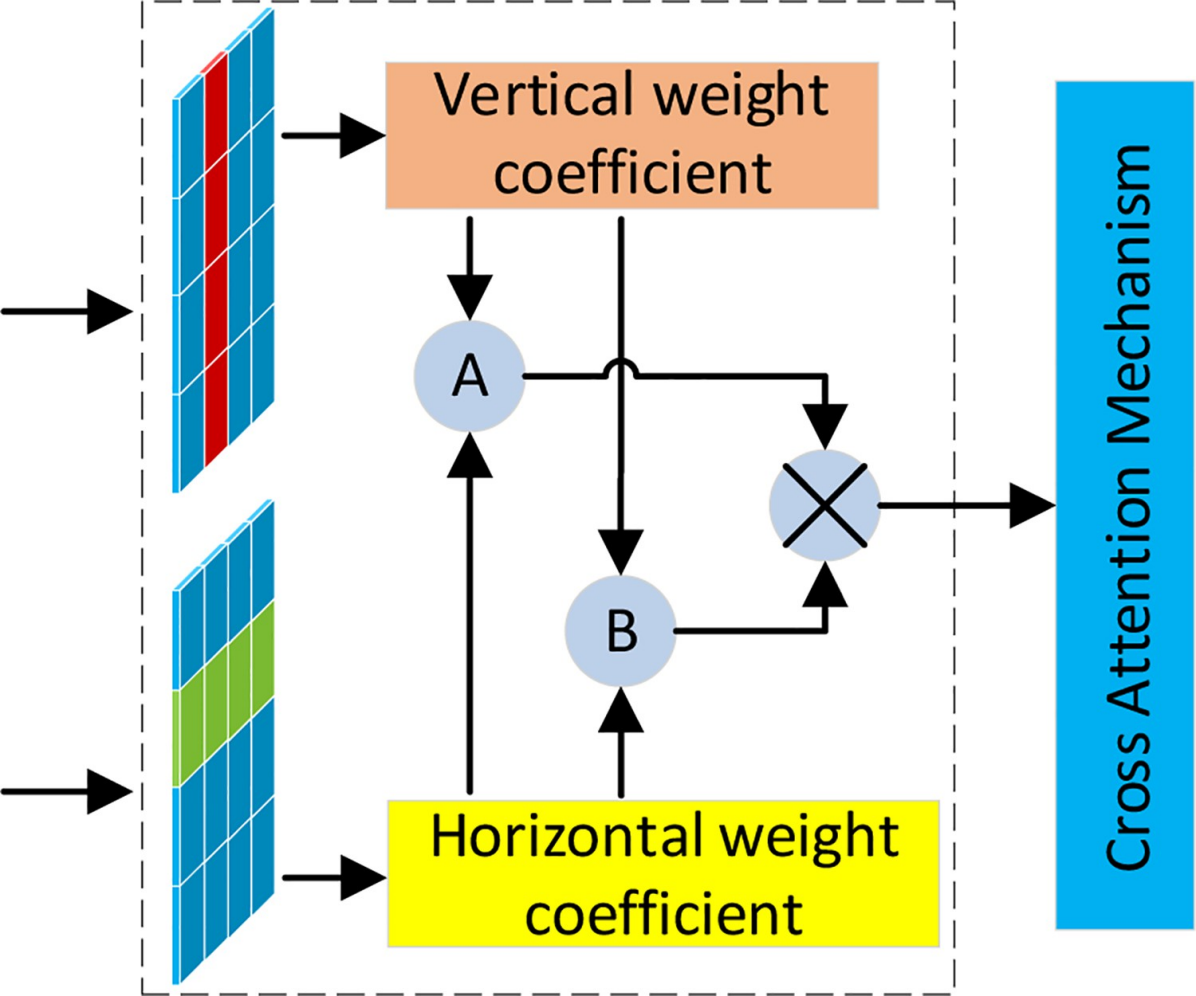
Mechanism of the cross-attention mechanism.

The cross-attention mechanism consists of three sections [25]. Section one is a type of attention mechanism that produces weight characteristics in the horizontal and vertical directions. Considering section two, two types of weight characteristics are multiplied to further expand the weighting coefficient (e.g., the small weight may be 0.1*0.2, whereas the large weight may be 0.9*0.7). Regarding section three, the two types of weight characteristics are matched to acquire the maximum value. This is used to replenish the weighting coefficient result of section two (e.g., *max* (0.1, 0.5). The weights of the three sections are spliced and fused as follows:

$$C_{i}=\sum_{j=1}^{n}\frac{\exp(e_{i,j})}{\sum_{k=1}^{n}\exp(e_{ik})}h_{j}$$ (4)

$${\mathrm{mul}=(c}_{I}{*c}_{\mathrm{II}})$$ (5)

$${\max=(c}_{I}{,c}_{\mathrm{II}})$$ (6)

$${\mathrm{CA}=\mathrm{concatenate}([c}_{I}{,c}_{\mathrm{II}},\mathrm{mul},\max]),$$ (7)

where *e*_*ij*_ represents the weighting coefficient assigned by the attention mechanism, and pixel *j* indicates the characteristic sequence. *i* refers to the characteristic of a matrix, and *h*_*j*_ denotes the hidden layer information *j* of the characteristic sequence. In addition, *c*_*I*_ represents the weighting coefficient of the vertical attention mechanism in the characteristic sequence (*c*_*I*_ = {*c*_1_, *c*_2_…*c*_*i*−1_, *c*_*i*_}), *c*_*II*_ is the weighting coefficient of the horizontal attention mechanism in the characteristic sequence (*c*_*II*_ = {*c*_1_, *c*_2_…*c*_*i*−1_, *c*_*i*_}), *mul* indicates the multiplication of weighting coefficients, and max denotes the maximum computed value of the weighting coefficient. Fig 5 shows a schematic diagram of the average cross-attention mechanism.

Considering Fig 5C1 and 5C2 represent the weighting coefficients in two directions, where A refers to the weight multiplication policy and B denotes the maximum weight policy. The weighting coefficient can be acquired by the Softmax function, expanded by the weight multiplication and maximum matching policies, and it can generate high-level characteristic through convolutional layer and mean pooling.

### 3.3 Model training

#### 3.3.1 Data preparation

Hardware environment: processor: Intel Core i9-10980XE, 500GB memory; graphics card: NVIDIA GeForce RTX 2080 TI; System Memory: 16G video memory.

Software environment: CUDA Toolkit 10.2.89, CUDA 10.2, Python 3.8, Pytorch-GPU 1.8, Operating system: Windows10.

The unified input size of pictures was 512 × 512, and a total of 409 pictures were captured. Based on the ratio of 8:2, the dataset was divided into training and test sets with 327 and 82 pictures for model training and testing, respectively.

#### 3.3.2 Training methods

**Fig 6:**
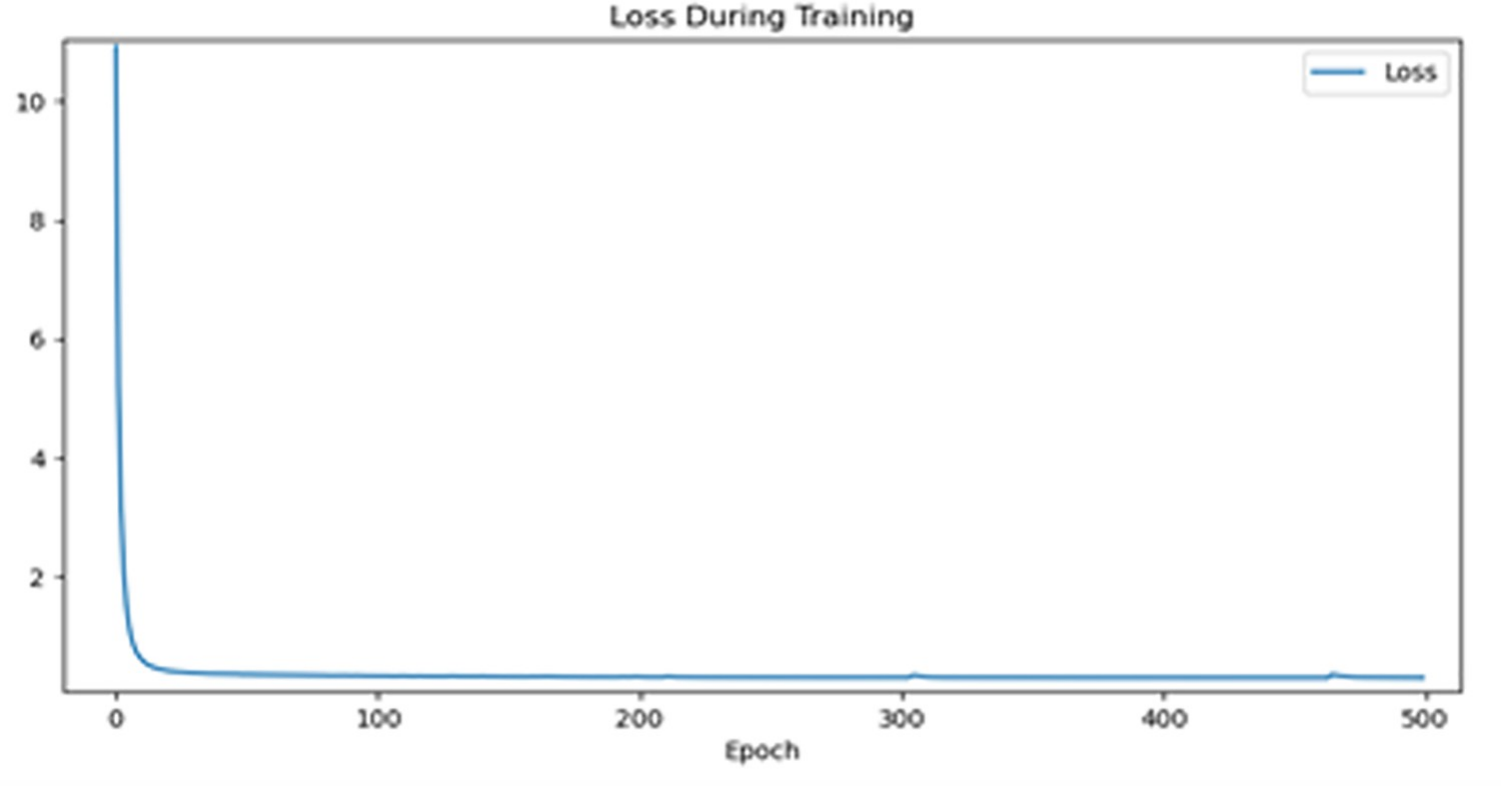
U-Net epoch-loss curves.

Considering the performance of hardware equipment and the training effect, the stochastic gradient descent (SGD) method was applied in this study for network training. Training with the Pytorch-GPU 1.8 framework in the environment of Python3.8 in the windows10 software environment, the training and test size of each batch was set to two, indicating that the batch size was two, and the momentum parameter was 0.9. Fig 6 shows the change in U-Net’s loss with epoch when the epoch is 500. When the number of epochs was 300, the loss tended to be stable. The epoch in this study was set to 300 because the setting of the learning rate will affect the convergence rate and stability of the model. Furthermore, when the callback function was added, the learning rate of the first 220 epochs was set as 0.0001, and that of the last 80 epochs was reduced ten times. Thus, the fitting speed can be increased, and the weight decay rate set to 0.0005.

Thanks to the use of the BEM block as the encoder of the BC-DUnet to the encoder of the decoder, making the BC-DUnet network a very strong fit, Within 100 epoch captures the feature information of the image with very high efficiency, the model quickly went to the fit state. In order to prevent the network model from overfitting or gradient disappearance, the DCFEM structure used by the BC-DUnet network can greatly prevent the overfitting of the network, and the number of feature graphs is constantly adjusted during the training process, transfer the feature mapping to all subsequent layers, overfitting is greatly prevented (Fig 6 is slightly adjusted accordingly when the epoch is about 305 and 470 times, respectively), in the following training, the network model can use more computation and learning to adjust the parameters.

## 4. Experimental results and analysis

### 4.1 Experimental evaluation criteria

Regarding the semantic segmentation, there are three most commonly used evaluation indexes, including pixel accuracy (PA), mean pixel accuracy (MPA), and mean intersection-over-union (MIoU) [26]. Pixel accuracy refers to the ratio of correctly labeled pixels to general pixels, MPA is the mean of all the classes obtained from the ratio of correctly classified pixels in each class, and MIoU indicates the proportion of intersection and union of the two sets: real and predicted values. Considering machine learning, another three indices Accuracy, Recall, and F1-Score are also normally used, among which Precision is the ratio of the actual crack pixels to the predicted crack pixels, and Recall refers to the ratio of the correctly predicted crack pixels to the actual crack pixels [27]. Furthermore, F1-Score considers both Precision and Recall, and can be considered as the weighted mean value of Precision and Recall. The expression for each index is as follows:

$$\mathrm{PA}=\frac{\sum_{i=0}^{k}p_{ii}}{\sum_{i=0}^{k}\sum_{j=0}^{k}p_{ij}}$$ (8)

$$\mathrm{MIoU}=\frac{\mathrm{TP}}{\left(\mathrm{FP}+\mathrm{FN}+\mathrm{TP}\right)}$$ (9)

$$\mathrm{MIoU}=\frac{1}{k+1}\sum_{i=0}^{k}\frac{p_{ii}}{\sum_{j=0}^{k}p_{ij}+\sum_{j=0}^{k}p_{ji}-p_{ii}}$$ (10)

$$\mathrm{Precision}=\frac{\mathrm{TP}}{\mathrm{TP}+\mathrm{FP}}$$ (11)

$$\mathrm{Recall}=\frac{\mathrm{TP}}{\mathrm{TP}+\mathrm{FN}}$$ (12)

$$\mathrm{FI}=\frac{2\times \mathrm{Precision}\times \mathrm{Recall}}{\mathrm{Precision}\times \mathrm{Recall}}$$ (13)

Where the true positive value (TP) refers to the number of pixels correctly recognized as cracks, and false positive value (FP) indicates the number of pixels mistakenly recognized as cracks. The false negative value (FN) denotes the number of pixels mistakenly recognized as non-cracks, and the true negative value (TN) represents the number of pixels correctly recognized as no cracks.

**Fig 7:**
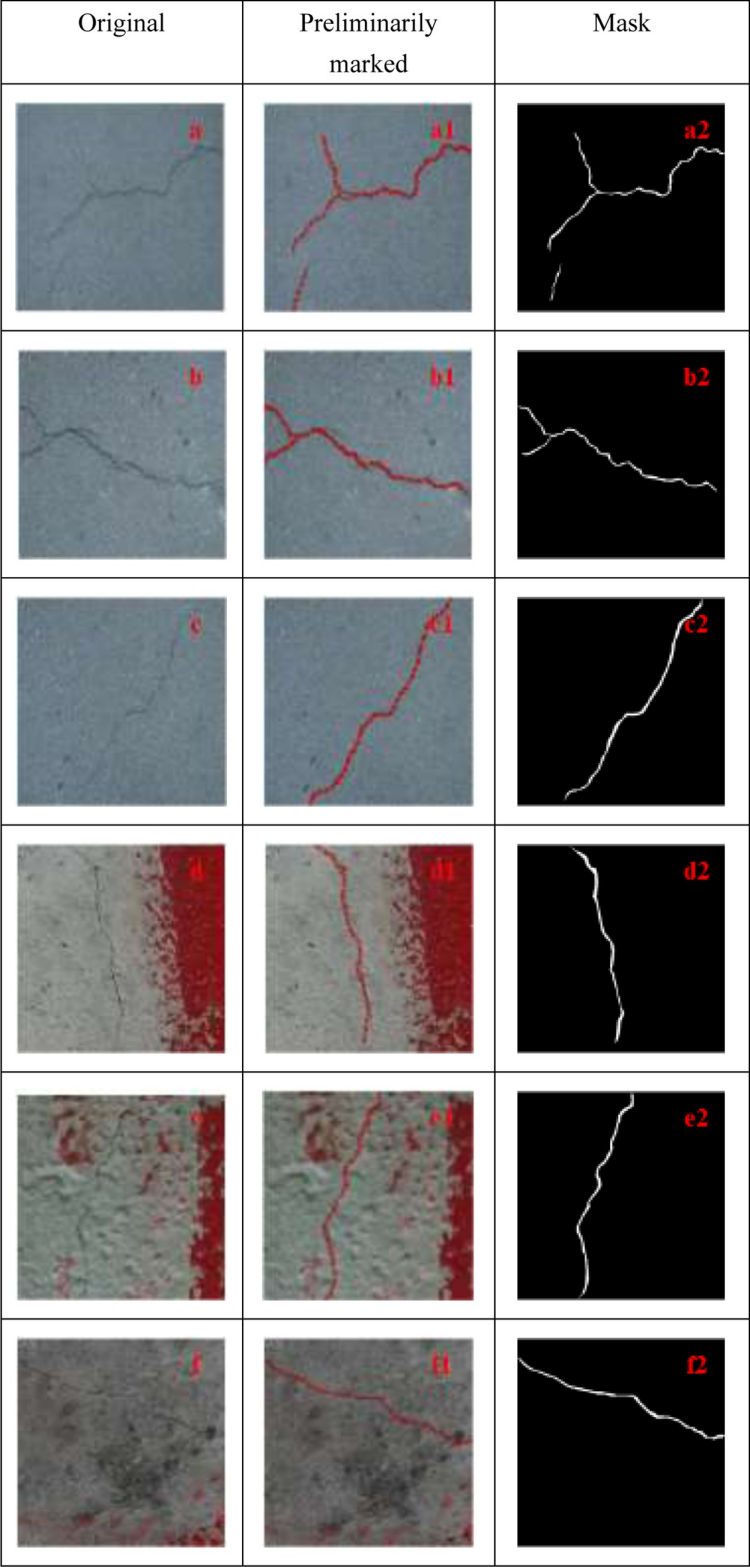
Sample of bridge crack markings.

Considering the data fitting process of U-Net, true standard data should be provided for the network. The weight of the model can be automatically adjusted through the loss value of the network predicted and actual results. Thus, the predicted value of the model is gradually approximated to the true value, and the tasks of bridge crack segmentation and detection can be completed. Therefore, the dataset was manually annotated. In this study, LabelMe, an image labeling tool, was applied for the manual annotation of each picture to acquire the segmentation label. The annotated files are saved in the JSON format, which mainly records the locations of annotation points and class labels. Fig 7 is the sample diagram of bridge crack markings.

Fig 7A–7F represent the original drawing of bridge cracks, respectively, among which Fig 7A–7C are the images of fine cracks in bridges, whereas Fig 7D–7F are pictures of bridge cracks with complex backgrounds. Fig d shows a picture of fine cracks with red stains, e is a picture of the bulged fine cracks with red stains, and f is a picture of sunken fine cracks with red stains. Furthermore, Fig 7a1, 7b1, 7c1, 7d1, 7e1, and 7f1, respectively represent the preliminarily marked images after the manual labeling, and Fig 7a2, 7b2, 7c2, 7d2, 7e2, and 7f2, respectively, are the mask maps. Black stands for background and white stands for bridge cracks.

**Fig 8:**
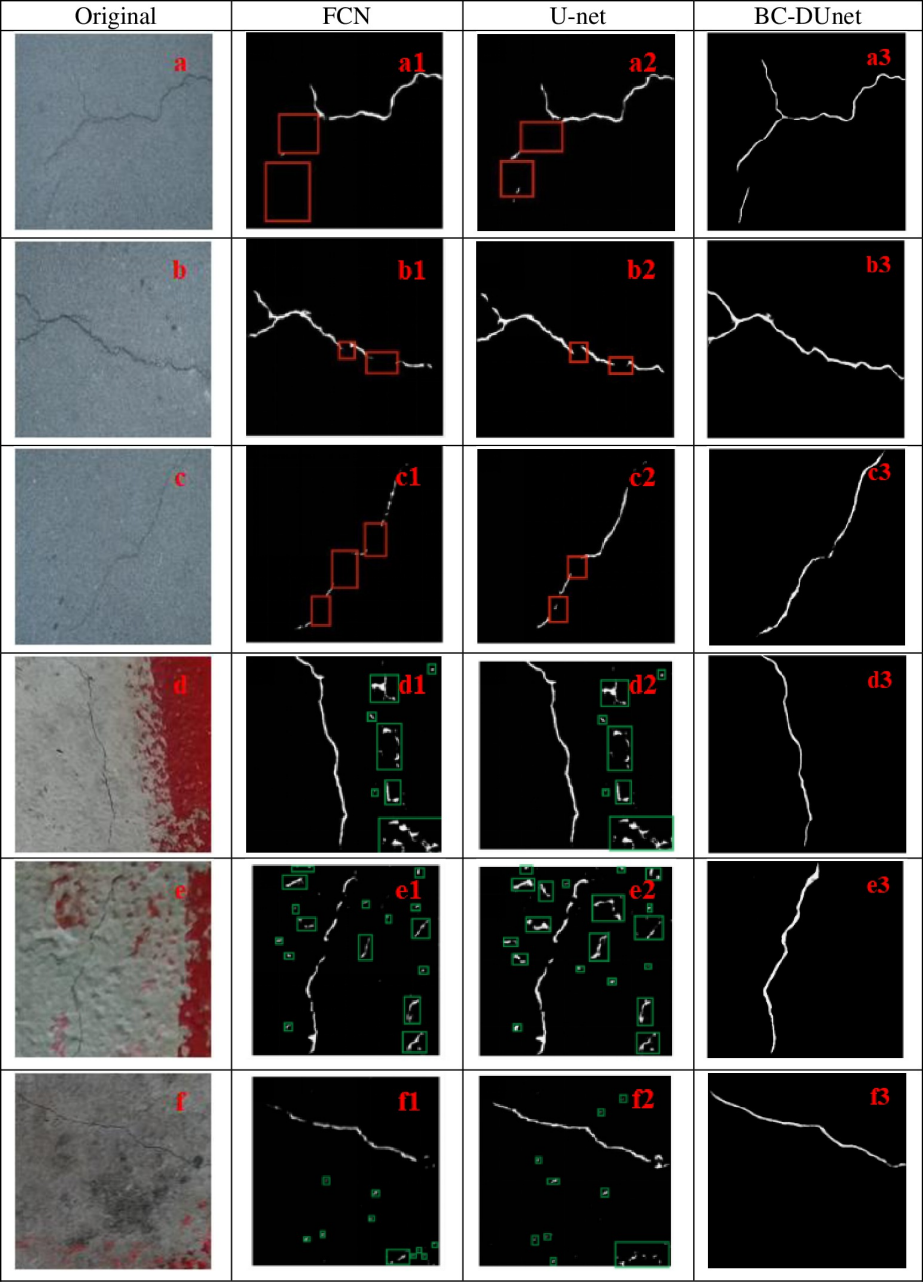
Comparison diagram of segmentation and segmentation effects pictures by FCN, U-Net, and BC-DUnet (Prop.) methods.

Fig 8 shows the segmentation effect by the FCN method, U-Net, and BC-DUnet (Prop.). The red rectangular box indicates the detection omission of crack pixels, and the green one represents the false detection of crack pixels. It can be observed from Fig 8 that the images in Fig 8A–8C are original drawings of fine cracks in bridges. Fig 8a1, 8b1, and 8c1 are segmentation effect pictures by the FCN method, Fig 8a2, 8b2, and 8c2 are segmentation effect pictures by the U-Net method, and Fig 8a3, 8b3, and 8c3 are segmentation effect pictures by the BC-DUnet (Prop.) method. As can be seen in these figures, the segmentation of fine cracks in bridges by FCN is not sufficiently precise, and it is not sufficiently sensitive to details. Compared to the FCN, U-Net significantly improves the segmentation effect of fine cracks. This is because the feature map acquired by each convolutional layer of U-Net will be spliced with the corresponding up-sampling layer. Therefore, the feature map of each layer can be effectively used in the subsequent calculation. Consequently, the precision of the model is improved to some extent; however, there are still missing crack pixels. The BC-DUnet can achieve an accurate segmentation of fine cracks.

Fig 8D–8F are the original effect pictures of fine cracks in bridges affected by the size of pavement texture particles and marking edge interference information. Fig 8d1, 8e1, and 8f1 are segmentation effect pictures by the FCN method. Fig 8d2, 8e2, and 8f2 are segmentation effect pictures by the U-Net method. Fig 8d3, 8e3, and 8f3 are segmentation effect pictures by the BC-DUnet (Prop.) method. It can be observed from these figures that it is difficult for the FCN and U-Net to achieve correct segmentation of bridge cracks with interference information, and the two methods cause false segmentation of both cracks and complex backgrounds. However, the BC-DUnet method proposed in this study can weight each feature channel based on the value of the feature image to improve the weight of the important characteristic and further promote the feature extraction effect. As shown in Fig d3, e3, and f3, the BC-DUnet can basically achieve accurate segmentation of bridge cracks.

To validate the ability of DUnet to extract crack details and the ability of BEM and CAM to extract crack information under redundant information, FCN, U-Net, and U-Net with characteristic modules were experimentally tested using U-Net as the baseline network in the same test environment. To verify that the BC-DUnet network has better performance than conventional, the experimental results were obtained in Table 2. BC-DUnet has 2.86% higher than FCN and higher IOU, and the rest such as Precision and Recall are higher than 8.12%, with BC-DUnet compared to FCN. Compared to U-net, the BC-DUnet improved by 4.04%, PA by 1.53%, BC-DUnet achieved 98.18%, and the rest such as Precision, Recall, F1-Score increased by no less than 4.83%. We can experimentally demonstrate excellent progress in the U-net-based improved BC-DUet.

**Table 2:** Experimental performance comparison table.

Network	PA	IOU	Precision	Recall	F1-Score
FCN	95.32%	46.73%	60.65%	69.72%	65.02%
U-net	96.65%	56.81%	63.32%	73.52%	69.49%
BC-DUnet	98.18%	60.85%	68.86%	81.28%	74.32%

### 4.2 Ablation experiment

**Fig 9:**
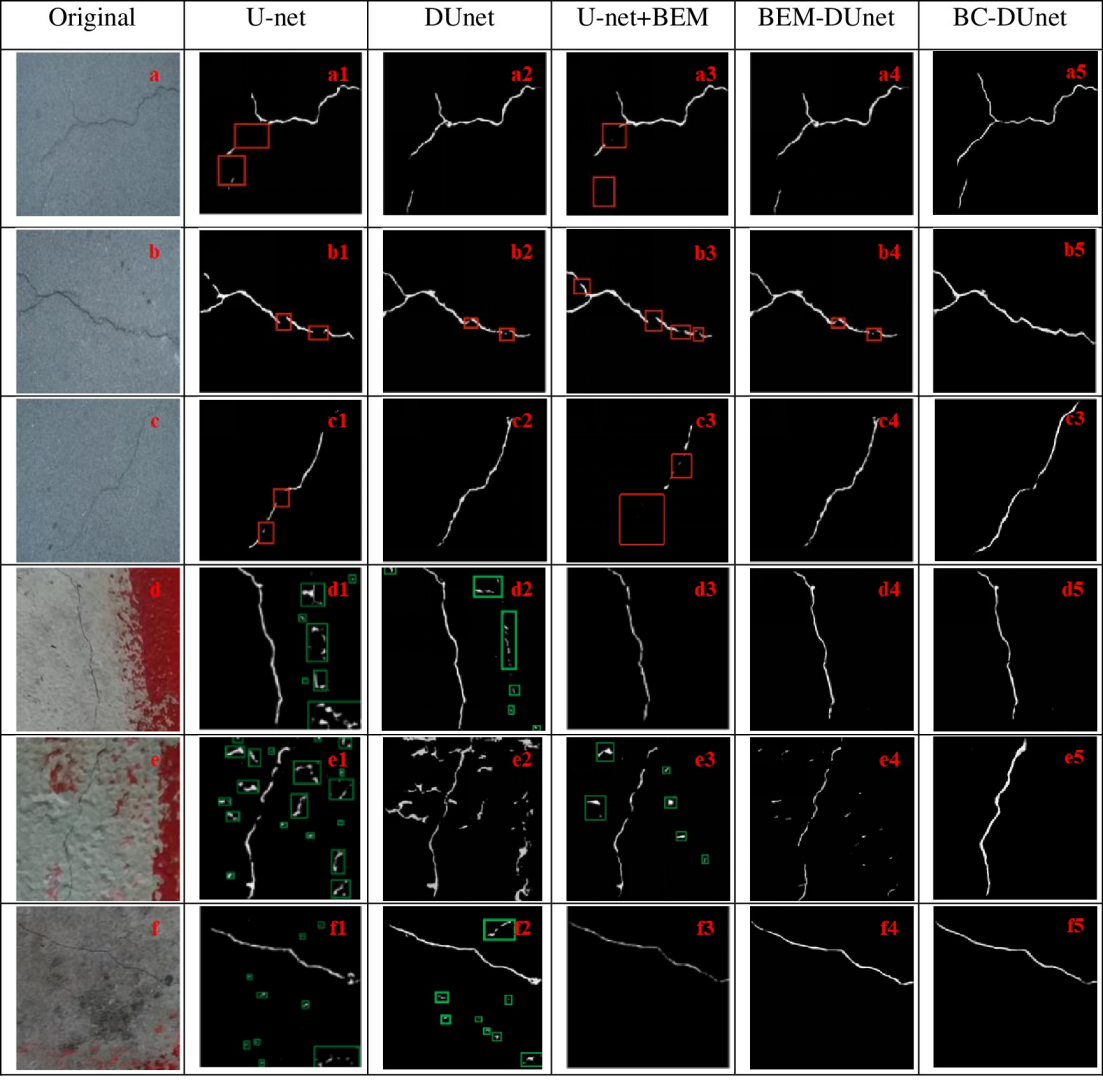
Ablation experiments.

Considering the bridge crack picture shown in Fig 7, an ablation experiment is performed using the method proposed herein. Fig 9 shows the segmentation effect pictures of bridge cracks by U-Net and DUnet(prop.), U-net+BEM(prop.), and DCFEM +BEM+CAM(Prop). The red rectangular box indicates the detection omission of the crack pixels, whereas the green box represents the false detection of the crack pixels. U-Net cannot segment fine cracks well, and there are many isolated and scattered points. Therefore, the segmentation details need to be strengthened. Moreover, the segmented crack boundary was not sufficiently refined. After the DCFEM is added, the segmented crack boundary is obvious, and a segmentation result similar to that of the segmentation label can be achieved. In addition, fine cracks can be segmented well, with few detection omissions, and the corresponding recall is also improved. Regarding the bridge crack images under complex backgrounds, although the FCN and U-net can be employed to recognize and segment cracks, they are much inferior to the method with the added BEM, considering noise removal. After the BEM module is added, the noise can be removed significantly, and the crack skeleton can be recognized and segmented completely and accurately.

Fig 9A–9C shows fine cracks in bridges. Fig 9a1, 9b1, and 9c1, are segmentation effect pictures by the U-Net method. Fig 9a2, 9b2, and 9c2, are segmentation effect pictures by DUnet(prop.). Fig 9a3, 9b3, and 9c3, are segmentation effect pictures by the U-Net+BEM (prop.). Fig 9a4, 9b4, and 9c4, are segmentation effect pictures by the BC-DUnet (DCFEM +BEM). Considering Fig 9a2, 9b2, and 9c2, DUnet achieves a better segmentation effect for fine cracks compared to the U-net. This is because the addition of the skip connection module facilitates the fusion of shallow and deep characteristics of the network. It effectively solves the vanishing gradient problem caused by excessive number of network layers, and further improves the fine crack segmentation progress of the network model. Moreover, it can be observed from Fig 9a3, 9b3, and 9c3, that because the background information of Fig 9A–9C, is relatively simple, after BEM is added to the baseline network U-Net, the crack segmentation effect does not change to a great extent. In fact, it does not improve the segmentation effect of fine cracks significantly. Furthermore, as shown in Fi 9a4, 9b4, and 9c4, the effective combination of DCFEM+BEM and U-Net can achieve accurate segmentation of fine cracks. Fig 9D–9F are the original drawings of fine cracks in bridges affected by the size of pavement texture particles and marking edge interference information. Moreover, Fig 9d1, 9e1, and 9f1, are the segmentation effect pictures by the U-Net method, and Fig 9d2, 9e2, and 9f2, are the segmentation effect pictures by U-Net + DCFEM (prop.). Fig 9d3, 9e3, and 9f3, are the segmentation effect pictures by U-Net + BEM (prop.). Fig 9d4, 9e4, and 9f4, are the segmentation effect pictures by DUnet+BEM (Prop). As shown in Fig 9d2, 9e2, and 9f2, U-Net causes false segmentation to different extents for prominent bridge decks and pits, and the addition of skip connection to the U-Net significantly improves the segmentation effect of the model for fine cracks; however, this method still leads to false segmentation of interference information. In addition, considering Fig 9d3, 9e3, and 9f3, after BEM is added to U-Net, the model can compensate for different scale features and increase the weight of important features. Although there are still missing pixels in the segmentation of fine cracks, the segmentation of crack skeleton can be realized under interference information. Furthermore, regarding Fig 9d4, 9e4, and 9f4, the effective combination of DUnet+BEM and U-Net can achieve complete segmentation of fine cracks under interference information, demonstrating the effectiveness of the method proposed in this study.

Fig 9a5, 9b5, 9c5, 9d5, 9e5, and 9f5, are crack detection effect diagrams after cross attention is introduced. Considering the figures, after cross attention is introduced, the network model achieves more precise segmentation effect for cracks, and there is no more obvious breakpoint compared yo the models as shown in Fig 9b4, 9e4, and 9f4. Compared to the models shown in Fig 9 a4, and 9c4, the models shown in Fig 9a5, and 9c5, can perform more complete crack segmentation.

Table 3 shows the experimental performance indexes of U-Net, DUnet (prop.), U-net+BEM (prop.), and BC-DUnet (prop.). After the DCFEM was added, the segmentation degree of the network to the fine cracks was strengthened, and the PA, IOU, and Precision were significantly improved compared to the U-Net. After BEM was added, the network enhanced the ability to extract important features; moreover, it extracted useful information in a complex background environment. Therefore, compared to the U-Net, the PA, IOU, Precision, and other indicators of the model with BEM added were improved. After the DCFEM and BEM were added, the model could enhance the segmentation effect on fine cracks in complex backgrounds and it performed complete segmentation of fine cracks, improving the accuracy of segmentation of fine cracks under complex backgrounds. It is superior to the DUnet and BEMD-Unet regarding all indexes. After DCFEM, BEM, and CAM were added, the proposed BC-DUnet could be used to segment fine cracks in a complex background.

**Table 3:** Experimental performance comparison table.

Network	PA	IOU	Precision	Recall	F1-Score
U-Net	96.65%	56.81%	63.32%	73.52%	69.49%
DUnet	97.72%	58.82%	66.44%	75.52%	72.32%
BEM-Unet	97.52%	59.02%	65.42%	74.32%	71.65%
BEMD-Unet	97.99%	59.87%	67.32%	78.82%	73.02%
BC-DUnet	98.18%	60.85%	68.86%	81.2%	74.32%

### 4.3 Comparison of the attention mechanism

**Fig 10:**
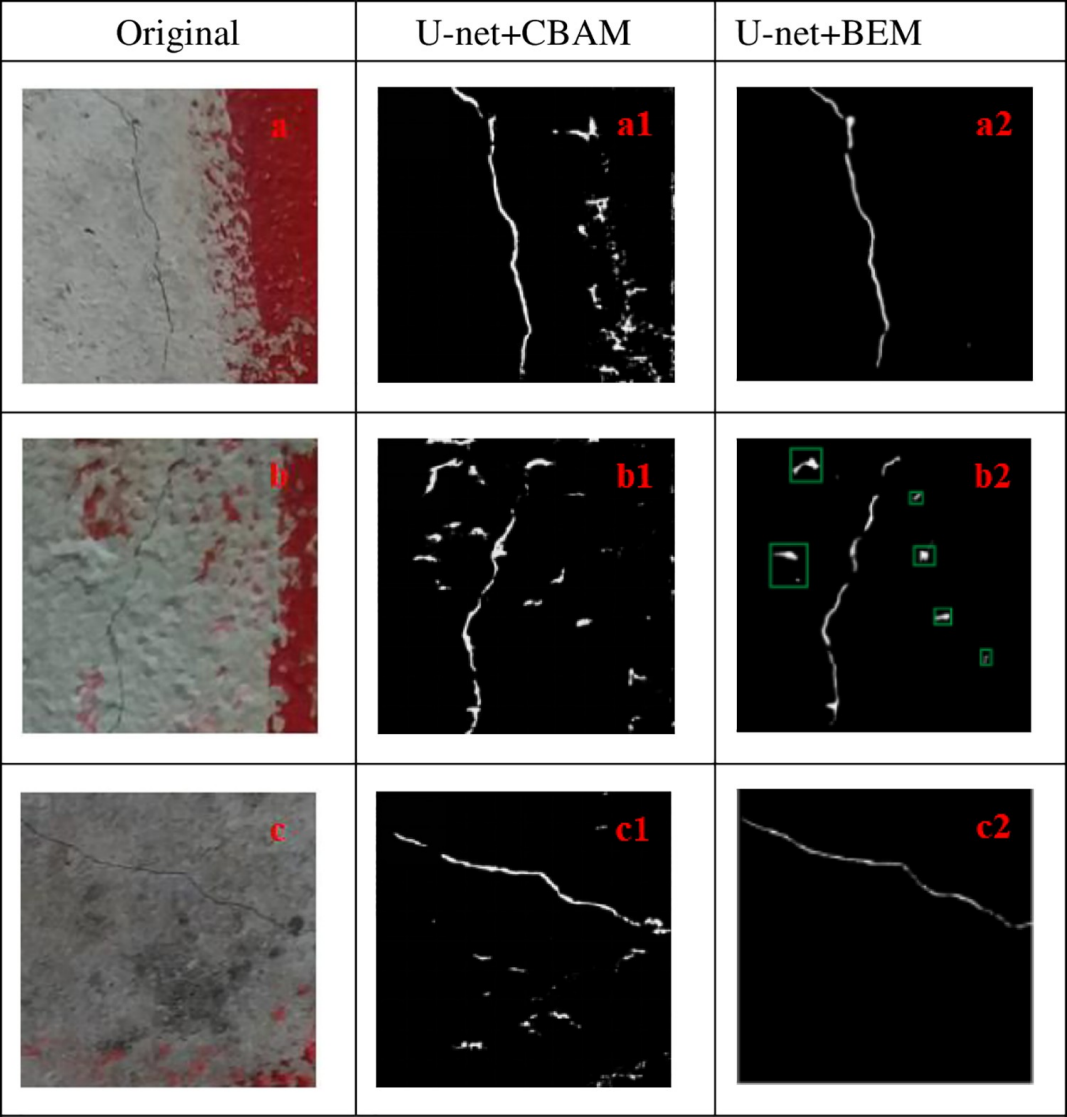
Attention mechanism comparison diagram.

To demonstrate the effectiveness of the attention mechanism proposed herein, it is compared to the classical attention mechanism, CBAM. Fig 10 shows the comparison results of the mechanism proposed herein and the U-net+CBAM method. Fig 10A–10C are the original effect pictures of fine cracks in bridges affected by the size of pavement texture particles and marking edge interference information. Fig 10a1, 10b1, and 10c1 are the segmentation effect pictures by the U-Net method, and Fig 10a2, 10b2, and 10c2 are the segmentation effect pictures by U-net + CBAM method; Fig 10a3, 10b3, and 10c3 are the segmentation effect pictures by U-net + BEM(prop.). As shown in Fig 10a2, 10b2, and 10c2, after CBAM is added to U-Net, the segmentation effect for fine cracks under the interference information is not significantly improved compared to the U-Net.

Nevertheless, U-net + BEM (prop.) can be employed for accurate segmentation of fine cracks under interference information, which further indicates that the attention mechanism proposed in this study can achieve an effective segmentation of the skeleton of fine cracks under interference information.

### 4.4 Comparison with other methods

To more accurately evaluate the performance of the BC-DUnet model, FCN-4s, CrackSegNet, and HU-ResNet were reproduced, respectively, and trained using a self-made dataset, and the experimental results are presented in Table 4. BC-DUnet leads the three networks, while BC-DUnet’s IOU is about 0.47% below HU-ResNet, BC-DUnet leads FCN-4s by about 9.84% in Precision values, thanks to the introduction of BEM modules, BEM eliminates the complex background affecting accuracy and greatly improves BC-DUnet Precision values. However, the maximum Recall difference of BC-DUnet reached 16.83% and a minimum difference of 3.04%.BC-DUnet has F1-Score values similar to CrackSegNet, but is about 0.31% ahead of CrackSegNet. Moreover, it is experimentally proved that thanks to BC-DUnet, BC-DUnet generalization better than all three networks because of has DCFEM block.

**Table 4:** Experimental performance comparison table.

Network	PA	IOU	Precision	Recall	F1-Score
FCN-4s [21]	94.01%	49.54%	59.02%	64.45%	63.32%
CrackSegNet [14]	96.42%	58.74%	67.42%	76.72%	74.01%
HU-ResNet [20]	96.32%	61.32%	66.32%	78.24%	69.02%
BC-DUnet (Prop.)	97.02%	60.85%	68.86%	81.28%	74.32%

## 5. Conclusion

1. In this study, the concept of semantic segmentation in deep learning is applied to bridge crack detection. This is improved based on the DCFEM, BEM, and CAM, and the BC-DUnet model is established. The model, based on U-Net, adopts the residual skip connection structure of DCFEM for deep feature extraction. This combines shallow detail information, deep semantic information, and BEM at the decoding stage, strengthening crack features and reducing the influence of interference information.
2. Considering the analysis of the contrast experiment with the FCN and U-Net network models, the BC-DUnet model is superior to other semantic segmentation models, considering the Precision, Recall, and F1-Score. Its detection performance for changes in fine cracks in bridges under interference information can meet the production demand; it is quite advantageous in improving the detection precision and acquiring more accurate detection results. It is highly accurate and has high generalization ability in the detection of fine cracks in bridges with interference information. Therefore, this network model can be applied to large-scale and visual dynamic detection of bridge cracks, providing a basis and theoretical method for urban planning and hazard assessment.
3. Deep learning and BC-DUnet-based crack detection still have certain restrictions regarding the use of images. As one of the most effective supervised learning methods, the BC-DUnet-based method requires an annotated crack dataset for training. However, achieving high-quality labeled crack segmentation images is laborious and time-consuming. Furthermore, crack pixels in image coordinates cannot be converted to physical or world coordinates owing to lack of camera calibration and scale factor for two-dimensional images. Therefore, it is difficult to acquire the physical parameters of cracks, such as the length, width, and area. Nevertheless, precise crack detection of images can further the multi-dimensional data fusion of images with other high-accuracy information, such as radar, laser, and GPS data, to compensate for the shortcomings of distinct information molds from multi-modal sensors. It also facilitates subsequent quantitative and refined detection of crack information.
